# FISH Amyloid – a new method for finding amyloidogenic segments in proteins based on site specific co-occurence of aminoacids

**DOI:** 10.1186/1471-2105-15-54

**Published:** 2014-02-24

**Authors:** Pawel Gasior, Malgorzata Kotulska

**Affiliations:** 1 Institute of Biomedical Engineering and Instrumentation, Wroclaw University of Technology, 50-370 Wroclaw, Poland

**Keywords:** Machine learning, Amyloid, Intramolecular contact sites, Hot spot

## Abstract

**Background:**

Amyloids are proteins capable of forming fibrils whose intramolecular contact sites assume densely packed zipper pattern. Their oligomers can underlie serious diseases, e.g. Alzheimer’s and Parkinson’s diseases. Recent studies show that short segments of aminoacids can be responsible for amyloidogenic properties of a protein. A few hundreds of such peptides have been experimentally found but experimental testing of all candidates is currently not feasible. Here we propose an original machine learning method for classification of aminoacid sequences, based on discovering a segment with a discriminative pattern of site-specific co-occurrences between sequence elements. The pattern is based on the positions of residues with correlated occurrence over a sliding window of a specified length. The algorithm first recognizes the most relevant training segment in each positive training instance. Then the classification is based on maximal distances between co-occurrence matrix of the relevant segments in positive training sequences and the matrix from negative training segments. The method was applied for studying sequences of aminoacids with regard to their amyloidogenic properties.

**Results:**

Our method was first trained on available datasets of hexapeptides with the amyloidogenic classification, using 5 or 6-residue sliding windows. Depending on the choice of training and testing datasets, the area under ROC curve obtained the value up to 0.80 for experimental, and 0.95 for computationally generated (with 3D profile method) datasets. Importantly, the results on 5-residue segments were not significantly worse, although the classification required that algorithm first recognized the most relevant training segments. The dataset of long sequences, such as sup35 prion and a few other amyloid proteins, were applied to test the method and gave encouraging results. Our web tool *FISH Amyloid* was trained on all available experimental data 4-10 residues long, offers prediction of amyloidogenic segments in protein sequences.

**Conclusions:**

We proposed a new original classification method which recognizes co-occurrence patterns in sequences. The method reveals characteristic classification pattern of the data and finds the segments where its scoring is the strongest, also in long training sequences. Applied to the problem of amyloidogenic segments recognition, it showed a good potential for classification problems in bioinformatics.

## Background

Amyloids are proteins which aggregate into oligomers and then fibrils that accumulate in cells. Their intramolecular contact sites form a characteristic zipper pattern. Although a few functional amyloids are known, the majority of proteins lose their physiological function when they aggregate and they become cytotoxic for cells [1-5]. The exact reason for this cytotoxicity is still unclear but many studies show that intermediate oligomeric structures are the main culprits. The number of amyloidogenic diseases following misfolding of a protein into the amyloid is constantly increasing and include Alzheimer’s disease (amyloid-β, tau), Parkinson’s disease (α-synuclein), type 2 diabetes (amylin), Creutzfeldt-Jakob’s disease (prion protein), Huntington’s disease (huntington), amyotrophic lateral sclerosis (SOD1), and many others (for a review see e.g. [6]). They affect constantly increasing number of people, especially in well developed countries. Recognition of factors responsible for protein misfolding can contribute to better understanding of its mechanisms and potential drug design. Recent studies indicate that there may be certain protein sequence determinants responsible for their affinity to form amyloids. These may be short segments of aminoacids, which are called hot spots [7,8]. Those fragments are harmless only when they are buried inside a protein. The amyloidogenic fragments responsible for amyloidogenicity of the whole protein are believed to be 4-10 residues long and it is often assumed that 6-residue fragments of amyloidogenic properties are typical “hot spots” [9]. Recognition of amyloidogenic fragments can be obtained by computational approach, for example physico-chemical methods, e.g. Tango [9], ZipperDB [10,11], FoldAmyloid [12,13], Pasta [14,15], AggreScan [16], PreAmyl [17], Zyggregator [18], CamFold [19], NetCSSP [20], AmyloidMutant [21,22], BetaScan [23], and consensus AmylPred [24]. Statistical methods have also been employed in the classification. In our previous work we used classical machine learning methods [25] implemented in WEKA [26]. Other methods include Waltz [27] using Position Specific Scoring Matrices (PSSM), or Bayessian classifier and weighted decision tree applied to long sequences of bacterial antibodies [28]. A few hundreds of amyloid peptides have been experimentally found, although the dataset is very limited. Also computational methods generate databases of potential amyloids, such as 3D profile [9,29], which is a physicochemical method that generated the most numerous computational dataset – ZipperDB [30].

In this manuscript we propose a new machine learning method for the identification of amyloidogenic segments in amino acid sequences, based on the presence of a segment with the highest scoring for co-occurrence of residue pairs. By application of a sliding window, the algorithm all by itself recognizes the most relevant training segments in positive training instances.

## Methods

### Machine learning method

Our classification method is based on the assumption that aminoacid sequences (such as amyloidogenic fragments) exhibit certain, well defined, pattern of residue distribution, which is position specific and, most importantly, involves co-occurrence of two aminoacids at different positions. For example, the pattern would not only include a high chance of valine occurrence at position 2, but also the valine would entail isoleucine at position 4. The pattern should be contained in one segment and limited in length. A pattern in the negative dataset is not important, as long as it is different from that of the positive set. However, it may happen that the discriminative pattern is more pronounced in the negative set - we also test our method with this regard. To investigate the co-occurrence pattern, a relevant window length needs to be specified. This window is equivalent to the minimal fragment of a protein sequence displaying the classification property.

**Figure 1:**
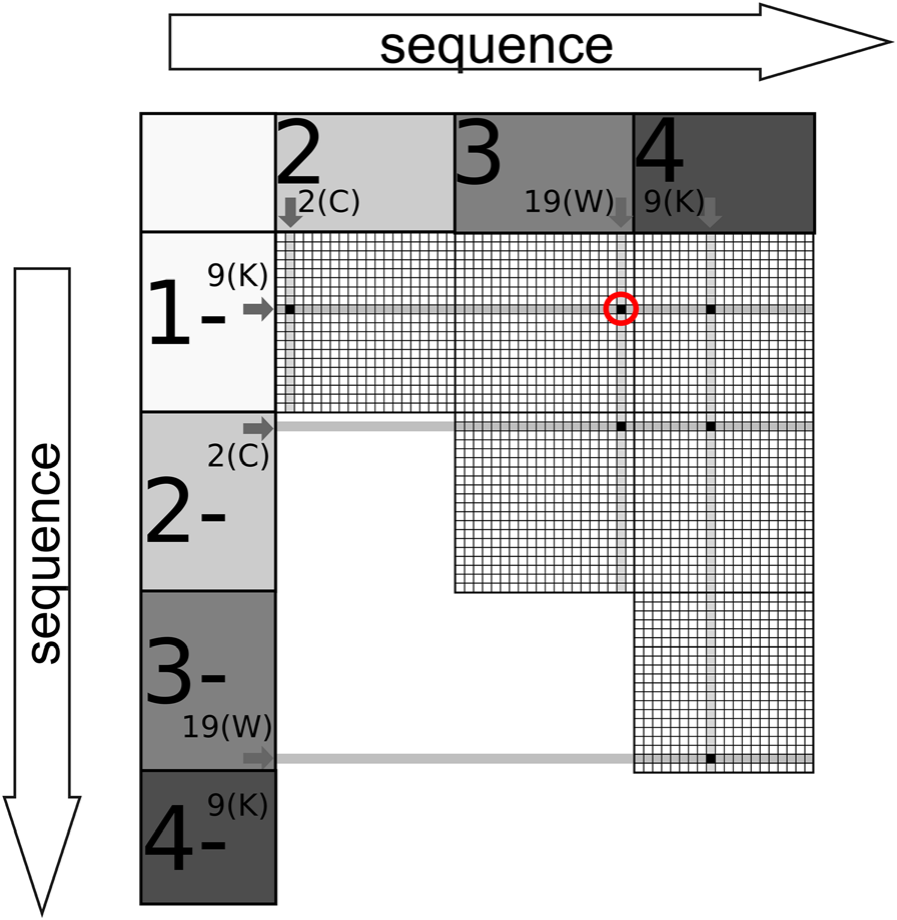
**Construction of the co-occurrence matrix.** Construction of the co-occurrence matrix (for the simplicity windows are of length 4, and 3 sub-matrices are generated in each direction of the general matrix). Coordinates of the general matrix (large numbers) represent the location of aminoacids in the sequences. Each aminoacid is represented by a number between 1 and 20 (ordered alphabetically), located within sub-matrices. For example, the point highlighted in red would indicate a high co-occurrence score between lysine (K) at position 1 of the sequence and tryptophan (W) at position 3 of the sequence.

First, the negative training dataset (*NO*) is divided into segments of the selected length *n* (here 5 or 6) by shifting the window of one position each time. We assume that there is no special segment in peptides from the negative dataset. Therefore, all generated negative segments equally contribute to their representative pattern and calculation of the classification threshold (described later in this section) used for discrimination between negative and positive test sequences. If the negative instances exhibit a pattern, it will be naturally averaged, hence removed, due to the shifting window. Pairs of aminoacids from all the segments are counted in the matrix *MatrixNO* (the explanation of co-occurrence matrices is presented in Figure 1), which represents occurrence of specific aminoacid couples with regard to their positions in the segments.

**Figure 2:**
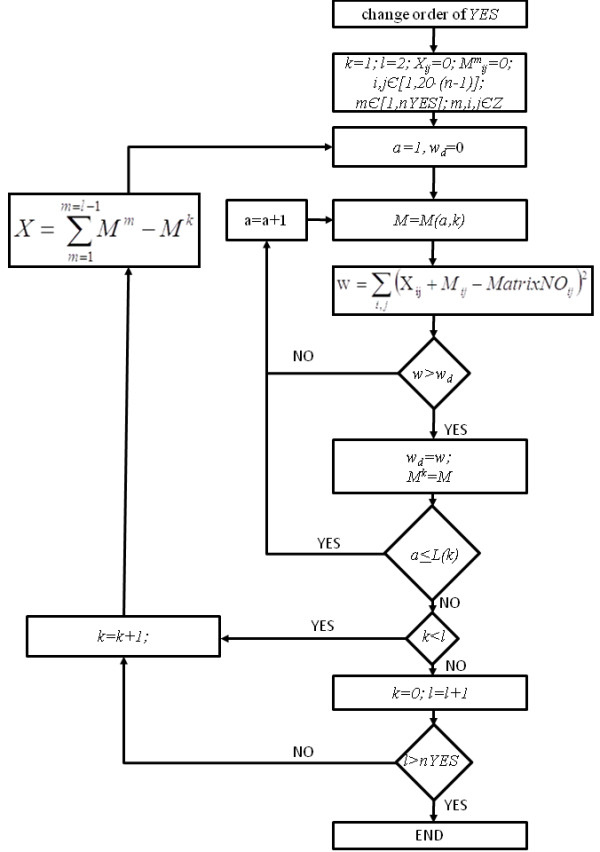
**Training algorithm.** Training algorithm of the method. Here *YES* (*NO*) denotes the set of positive (negative) training sequences, including *nYES* (*nNO*) number of instances, which are tested with a window of a length *n*; *MatrixYES* (*MatrixNO*) are corresponding co-occurrence matrices with coordinates *i* and *j*; *k* denotes the subsequent number of a positive training sequence, *M*_*k*_ is a temporary positive correlation matrix obtained up to the *k*-th sequence, a denotes the beginning position of a tested window; *X* is the normalized sum of all previously calculated matrices *M*; *l* is an iteration counter; w denotes distance between current positive and negative co-occurrence matrices, *w*_*d*_ is the maximal distance later used in the classification.

Next, the dataset of positive training instances (*YES*) undergoes similar procedure, generating *MatrixYES*. However, in contrast to the negative training instances, each positive training sequence can include segments responsible for amyloidogenicity of the sequence, whose location is not known, as well as segments lacking the pattern. Our method finds and takes into account only those segments which display the classification co-occurrence pattern in the most pronounced way, neglecting others. Hence, only one window (e.g. with the highest chance of amyloidogenicity) is selected in each positive training sequence, and each positive training sequence contributes only one segment to *MatrixYES*. Graphical representation of the final matrix which is used in the classification is presented in Figure 2. The most frequent couples of aminoacids (represented by numbers 1-20), from the selected 5-residue windows, assume the darkest color of the dot.

The most relevant segments in positive training sequences, carrying the classification pattern, are found in the iterative procedure that selects those which are most distant from the averaged pattern of negative segments, as well as closest to the segments selected from other positive sequences. The distance, *w*, between positive and negative segments is represented by a sum of elements of array *MatrixYES* divided by *MatrixNo*. The procedure, resulting with the choice of optimal segments in the set of positive training fragments, gives the maximum distance value, *w*_*d*_, which is used in the classification procedure as a threshold value.

In the classification of test sequences, a distance *w*_*l*_ is defined, which is an a’priori assumed ratio of *w*_*d*_ (between 0 and 1), providing a threshold value used in the classification test of sequences. Detailed training algorithm of the method is presented in Figure 2. In the classification of the test set (or a set of unclassified sequences), the greatest actual distance ratio, *w*_*s*_, between *MatrixYES* of the tested sequence and *MatrixNO* is calculated. If *w*_*s*_ assumes a value greater than a selected value of *w*_*l*_ then the window is classified as positive (Figure 2).

The overall quality of the classifier was evaluated with Area under Receiver Operating Characteristic (ROC) curve (AUC ROC). The value of the AUC ROC can range from zero to one, with the score of 0.5 corresponding to random guess and the score of 1 indicating perfect separation. Two methods of testing our machine learning method were applied: either the same set was used for training and validation or the method was trained on one dataset and tested on another one. In the first case, 4-fold cross-validation method was used and the mean result of AUC ROC was reported. Additionally, for evaluation of the method, we used Sensitivity (*Sn*), which is the ratio of correctly classified positive fragments and Specificity (*Sp*), the ratio of correctly classified negative fragments. They are defined in the following:

$$\begin{array}{l} \mathrm{Sn}=\mathrm{TP}/\left(\mathrm{TP}+\mathrm{FN}\right)\\ \mathrm{Sp}=\mathrm{TN}/\left(\mathrm{TN}+\mathrm{FP}\right) \end{array}$$

where TP, FP, FN and TN represent the numbers of true positives, false positives, false negatives and true negatives, respectively.

### Datasets

Our classification method was first trained and validated on 3 experimental datasets of short peptide fragments, specifying their amyloid or β-aggregation propensities: AmylHex [9] with 6-residue sequences including 67 positive and 91 negative, Waltz [27] with 6-residue sequences including 49 positive and 71 negative, Tango (TG, tested for aggregation) [9] with a variable (4-43) residue fragments including 71 positive and 172 negative instances, downloaded from FoldAmyloid database [31]. The choice of experimental datasets is very limited since very few data are available, and our choice included all of them. Unfortunately, all these datasets are biased, which can influence the results of machine learning.

To compare the performance of our classification method with classical machine learning methods, we used another dataset of 4481 hexapeptides, which was computationally obtained with the 3D profile method [25]. The 3D profile method was originally proposed in [9] and applied in ZipperDB to generate the database of amyloidogenic hexapeptides. This computational dataset was generated with a faster version of the 3D profile algorithm [25]. It is not as biased as the experimental datasets and it was previously used in tests with a number of classical machine learning methods [25].

Then, our classification method, trained with 5-residue sliding window on the set of short peptides from Waltz dataset [33], was tested on 4 full length amyloidogenic proteins: amyloid-β and tau (Alzheimer’s disease), α-synuclein (Parkinson’s disease), amylin (type 2 diabetes), and prion protein sup-35 (Creutzfeldt-Jakob’s disease). The Waltz dataset was selected for the training since it did not contain fragments of the tested proteins. In these proteins, the method indicated amyloidogenic regions, classified with various values of the classification threshold *w*_*l*_, which were compared with experimentally validated data.

Finally, we merged all the experimental datasets. The full dataset included all experimentally tested peptides from different groups, whose length did not exceed 10 aminoacids, and involved also fragments from prion sup35. The full dataset consisted of 436 (146 positive and 290 negative) fragments (see Additional file 1). This dataset was first used in 4-fold cross-validation of our method, and then to train our web service *FISH Amyloid*, which is now freely available for classification.

## Results and discussion

Our method was trained on hexapeptides from different datasets, using two sliding window lengths: 5 and 6 (note that training on the 6-residue fragments with a window of length 6 eliminates the stage of finding the most relevant pattern-carrying, windows in the training and testing sequences). The results, obtained with different classification threshold *w*_*l*_ were represented as ROC curves.

Testing the quality of our new classification method and comparing it with different methods could only be possible while working with the same datasets as those state-of the-art methods. Therefore, to compare the performance of our method to classical machine learning methods, first we ran tests on the non-biased computational dataset generated with the physicochemical 3D profile method [9]. The result can be used for comparison with other machine learning methods since the same dataset was previously classified with several classical machine learning methods implemented in WEKA [25]. In this case, AUC ROC obtained with our method was 0.95 for a 6-residue window and 0.87 for a 5-residue sliding window. Top results of the state of the art methods from WEKA, working on hexapeptides, were very similar. For example, neural network (multilayer perceptron – MLP) and alternating decision tree, which showed the highest performance for this dataset from over 100 machine learning methods available in WEKA, obtained AUC ROC = 0.96 [25]. This is very similar to our results with the method presented here, obtained for the 6-residue window. Other classical methods implemented in WEKA obtained lower quality. Moreover, the result of new method was not significantly worse when it worked on a sliding window of length 5, although it first required that the algorithm finds the most relevant windows in the training and testing sequences. Hence, the classification quality of the new method presented here was very close to the top results obtained with classical machine learning methods on the same dataset. Moreover, none of the classical methods was capable of finding the most relevant training window, which is an asset of our new method.

Then, the performance of our method was tested on experimental datasets, which are scarce and possibly incompatible with each other. Hence, we first used those datasets separately. Depending on the applied experimental dataset, the AUC ROC varied from 0.69 to 0.81 for a sliding window of length 5 and between 0.69 and 0.79 for a window of length 6 (Table 1, main diagonals, bold font). Additionally, to test if negative datasets could have discriminative patterns, we ran the classifications in which the negative sets were treated as “positive”. The results are presented in Table 1 as a second number in each field, showing that many of those negative datasets are biased. Only the values close to 0.5 mean the lack of any characteristic pattern. By combining different datasets and testing one versus another, we could observe how compatible they are with each other (Table 1, non-diagonal). The AUC ROC values were lower in this case, showing that the available datasets are often incompatible.

**Table 1:** Classification results

**Training set (horizontal)**	**TG**	**Waltz**	**AmylHex**
**Tested set (vertical)**			
	**sliding window of length 5**		
**TG**	**0.75** | 0.62	**0.82** | 0.21	**0.77** | 0.42
**Waltz**	**0.62** | 0.60	**0.69** | 0.60	**0.59** | 0.51
**AmylHex**	**0.69** | 0.60	**0.84** | 0.31	**0.81** | 0.47
	**sliding window of length 6**		
**TG**	**0.76** | 0.57	**0.77** | 0.30	**0.78** | 0.44
**Waltz**	**0.54** | 0.45	**0.69** | 0.61	**0.61** | 0.43
**AmylHex**	**0.48** | 0.57	**0.82** | 0.25	**0.79** | 0.47

AUC ROC of the classification results with two window lengths. To test if a classification pattern is observable in the negative datasets, the training and testing procedures were also applied on negative datasets (POSITIVE | NEGATIVE); the positive datasets are in bold. Training dataset is defined horizontally; testing dataset – vertically. Random classification (or no pattern in a dataset) would obtain 0.5 and an ideal classifier 1.

The performance of our method was then compared to two state of the art tools for classification of amyloidogenic hot spots: Waltz, which was based on the most numerous individual dataset tested above, and FoldAmyloid using a combination of several experimental datasets. The authors of Waltz show [27, Addenum Figure 1] that their method trained on Waltz hexapeptides and tested on AmylHex dataset generated ROC curve with diagonal coordinates *Sn* = 83% and *Sp* = 83% (Waltz) and *Sp* = 89% and *Sn* = 89% (cross-validated Waltz), AUC ROC was not reported. Our method, trained on 5-residue sliding windows from Waltz dataset and tested on AmylHex obtained *Sn* = 79%, *Sp* = 78%, and AUC ROC = 0.81. Cross-validation of Waltz was reported at the level of *Sn* = 84%, *Sp* = 92% [27]. (Our method, in the more demanding mode i.e. with a sliding window of length 5, trained on the Waltz dataset, obtained AUC ROC of 0.69 and diagonal point of the ROC curve was *Sn* = 63% and *Sp* = 63%). However, an independent test on fragments from prion sup35 showed the adventage of our method. Waltz authors reported *Sn* = 58%, *Sp* = 90%, while our method (also trained on the Waltz dataset but with a 5-residue long sliding window) obtained *Sn* = 70% and *Sp* = 91% (Table 2). For comparison, the authors of Waltz also reported the sensitivity of computational 3D profile method on sup35 positive set, which was *Sn* = 67% [27].

**Table 2:** Tests on prion sup35 fragments

**Positive fragments**	**Classification result**	***w***_***s***_
7–17 GNN**QQNYQ**QY	+	0.34
16–26 Y**SQNGN**QQQG	-	0.08
28–38 R**YQGYQ**AYNA	+	0.21
43–53 GGY**YQNYQ**GY	+	0.53
46–56 **YQNYQ**GYSGY	+	0.53
52–62 **YSGYQ**QGGYQ	+	0.16
55–65 YQQGG**YQQYN**	+	0.13
94–104 PQGG**RGNYK**N	-	0.09
103–113 **NFNYN**NNLQG	+	0.22
106–116 YNNN**LQGYQ**A	+	0.17
109–119 N**LQGYQ**AGFQ	+	0.17
127–137 ND**FQKQQ**KQA	-	0.11
**Negative fragments**		
67-76 AG**YQQQY**NPQ	+	0.17
70-79 QQ**QYNPQ**GGY	-	0.08
73-82 YNPQG**GYQQY**	-	0.06
76-85 QGG**YQQYN**PQ	+	0.13
79-88 **YQQYN**PQGGY	+	0.13
82-91 YN**PQGGY**QQQ	-	0.03
139-148 KPKKT**LKLVS**	-	0.09
142-151 KT**LKLVS**SSG	-	0.09
145-154 KLVS**SSGIK**L	-	0.12
148-157 S**SSGIK**LANA	-	0,12
151-160 GIK**LANAT**KK	-	0,07
154-163 **LANAT**KKVGT	-	0,07
157-166 ATK**KVGTK**PA	-	0,03
160-169 K**VGTKP**AESD	-	0,03
163-172 TKPA**ESDKK**E	-	0,03
166-175 A**ESDKK**EEEK	-	0.03
169-178 DK**KEEEK**SAE	-	0.03
172-181 E**EEKSA**ETKE	-	0.03
175-184 KSAE**TKEPT**K	-	0.06
178-187 E**TKEPT**KEPT	-	0.06
181-190 EPT**KEPTK**VE	-	0.06
184-193 KEPTK**VEEPV**	-	0.09
187-196 TK**VEEPV**KKE	-	0.09
190-199 EEPVK**KEEKP**	-	0.03
193-202 VK**KEEKP**VQT	-	0.03
196-205 EEKPV**QTEEK**	-	0.03
199-208 PVQ**TEEKT**EE	-	0.11
202-211 **TEEKT**EEKSE	-	0.11
205-214 KTEEK**SELPK**	-	0.08
208-217 EK**SELPK**VED	-	0.08
211-220 ELP**KVEDL**KI	-	0.11
**Sensitivity**	**0.75**	
**Specificity**	**0.91**	

Classification efficiency tested on fragments of prion sup35. The method was trained on Waltz dataset with a sliding window 5-residue long, classification coefficient was set to *w*_*l*_ = 0.13. Emphasized are windows recognized by the classification method as potentially the most positive (amyloidogenic) of the whole tested fragment; the fragments obtained the actual distance value *w*_*s*_ (window of a greatest distance).

With the optimal parameters, FoldAmyloid was reported to obtain: for the scale of the expected packing density *Sn* = 75%, *Sp* = 74%, for the donor scale *Sn* = 69%, *Sp* = 78%, for the acceptor scale *Sn* = 0.77 and *Sp* = 74% [13]. Our method, trained on the same dataset as FoldAmyloid, with a 5-residue sliding window, obtained AUC ROC = 0.82, the diagonal point of the ROC curve was *Sn* = 75% and *Sp* = 75%.

**Figure 3:**
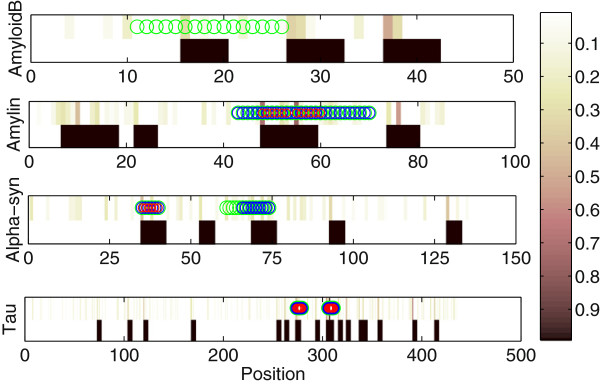
**Classification of long proteins.** The results of our classification on 4 amyloid proteins. The method was trained on Waltz dataset. Black blocks indicate location of amyloidogenic segments obtained with *w*_*l*_ = 0.14, which was equivalent to the specificity of 60% on Waltz dataset. The brown blocks at the top indicate where the amyloidigenic segments would begin if a different *w*_*l*_ value would be assumed. The circles show amyloidogenic segments obtained experimentally by different groups, working on protein fragments of various lengths (green – above 16, blue -11, red - 7).

We also tested our method on full length amyloid proteins. For all full protein independent tests we were using our method trained on hexapeptides from Waltz dataset, which does not include their fragments [27]. To apply a full version of our algorithm, with recognition of the most relevant windows in the positive training instances, we applied a window of length 5. Four full-length amyloid proteins were tested: amyloid-β, τ, amylin, and alpha-synuclein. The results are presented in Figure 3, where black blocks indicate location of amyloidogenic segments obtained with *w*_*l*_ = 0.14, which was equivalent to the specificity of 60% obtained on Waltz dataset with a cross-validation method. The brown blocks at the top of lines indicate where the amyloidigenic segments would begin if a different *w*_*l*_ value would be assumed. We compared the classification results to the experimental data. The circles show amyloidogenic segments obtained experimentally by different groups, working on protein fragments of various lengths. Amyloid-β: 13-24 HHQKLVFFAED, 11-26 EVHHQKLVFFAEDVG 48-53 [9], amylin: 48-53 FLVHSS, 55-60 NFGAIL [9]; Alpha-syn: 35-40 EGVLYV [27], 61-73 EQVTNVGGAVVTG, 66-74 VGGAVVTGV [27]; Tau: 274-279 KVQIIN [27], 306-311 VQIVYK [9,10,27].

The method was capable of finding most of the segments that have already been experimentally confirmed. It can be observed that other fragments have also been shown as potential hot-spots, however most of them have not been experimentally tested.

**Figure 4:**
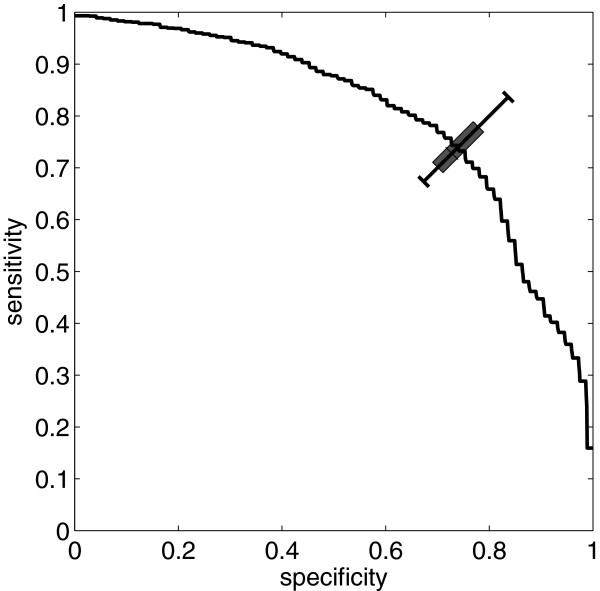
**Classification performance on a complete experimental dataset.** ROC obtained with FISH Amyloid on all available experimental data (all datasets with peptides 4-10 aminoacids long and experimental fragments from sup35). The total AUC ROC is 0.80 and the diagonal classification point has both *sensitivity* and *specificity* of 74%. The curve is based on average values of 40 independent trials from 4-fold cross-validations. The quantiles 0.95, 0.85 and median are presented as a boxplot at the diagonal classification point.

Finally, we merged all the experimental datasets to study the application of our method for practical recognition of the amyloidogenic sequences. The extended dataset contained all experimentally studied peptides of 4-10 aminoacids. Figure 4 presents the average-value ROC curve obtained with our method on this dataset from 40 independent trials by 4-fold cross-validation. The total AUC ROC was 0.80 and the optimal (diagonal) classification point had sensitivity *Sn* = 74% and specificity *Sp* = 74%. The quantiles of 0.95, 0.85 and median are presented as a boxplot at the diagonal classification point of the ROC curve.

Based on this extended experimental dataset, we trained our method for finding amyloidogenic windows in aminoacid sequences, and made it available as a web tool called *FISH Amyloid* (Hot Spot Is Found in Amyloid - reversed), which is currently available at http://www.comprec.pwr.wroc.pl/COMPREC_home_page.html. The service uses 5-residue sliding windows, both for training and classification, displaying the score value at the beginning of each window. Those residues that belong to at least one positive window are classified as positive and denoted by “1”. The list of fragments that constituted the extended dataset is also available at the service site.

The classification on the extended dataset was also compared with the performance of Waltz and FoldAmyloid (packing density) methods. Using 75% of data in each of 4 test, FoldAmyloid showed *Sn* = 58%, *Sp* = 75%, Waltz obtained *Sn* = 71%, *Sp* = 83%, and *FISH Amyloid* in the same 4 tests achieved *Sn* = 76% and *Sp* = 76% (see Additional file 1).

**Figure 5:**
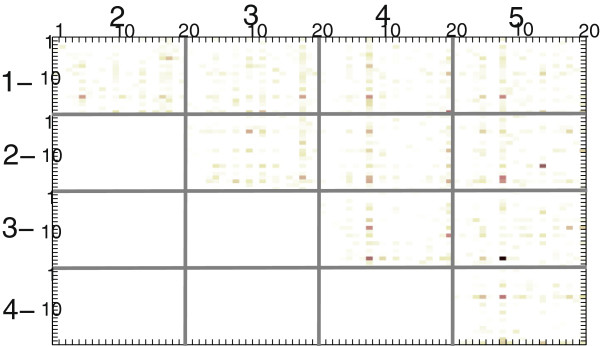
**Final co-occurrence matrix.** Graphical representation of the final co-localization matrix on extended experimental dataset. Large matrix coordinates represent the location of aminoacids couples, obtained from the 5-residue sliding window. The most frequent couples of aminoacids, which indicate the classification pattern, assume the darkest colors of dots. Aminoacids are denoted with small numbers, ordered alphabetically (A = 1, C = 2, D = 3, E = 4, F = 5, G = 6, H = 7, I = 8, K = 9,L = 10, M = 11, N = 12, P = 13, Q = 14, R = 15, S = 16, T = 17, V = 18, W= 19, Y = 20).

The most interesting feature of the method presented here is its ability to reveal a co-occurrence pattern found in the positive training dataset. The pattern includes pairs of aminoacids with their positions, which most frequently occur together. The patterns found in the full experimental dataset is presented in Figure 5. Table 3 shows the final pairs after executing the cut-off at the threshold of 0.4.

**Table 3:** Co-localized pairs of aminoacids

		**2**	**3**	**4**	**5**
All + sup35	**1**	S-F, G-V	S-V, Y-N	S-I, Y-Y, L-Y, I-I	S-I, Y-Q
	**2**		F-L, T-V	V-I, T-I, V-Y, F-I	R-R, T-I, V-I
	**3**			V-I, L-I, N-Y	V-I, L-V
	**4**				I-I, I-F, Y-R, Y-I

The co-occurrence pattern obtained with the sliding window of length 5 after training on extended experimental dataset. The first letter of each pair corresponds to the window location indicated by vertical numbers, the second letter to a number indicated horizontally. The table was obtained from data presented in Figure 5 after executing the cut off for the threshold of 0.4.

## Conclusions

We proposed an original classification method which recognizes classification pattern in sequences, taking into account position dependent frequency of aminoacids and site specific co-occurrence between their pairs. The method reveals the characteristic co-occurrence pattern of the data. Moreover, it is able to find the segments with the co-occurrence pattern of the highest scoring, also in long training sequences, and use them for the training. Our method was applied to the problem of recognition of amyloidogenic segments and it showed a good potential for their classification. We obtained good results for a sliding window of lengths 6 and 5. The web tool *FISH Amyloid*, using this method trained on full experimental dataset of amyloid fragments 4-10 aminoacids long, with 5-residue sliding window, is currently available at our server: http://www.comprec.pwr.wroc.pl/COMPREC_home_page.html (it will be moved to http://www.comprec.edu.pwr.wroc.pl/COMPREC_home_page.html). *FISH Amyloid* offers prediction of amyloidogenic segments in protein sequences.

## Competing interests

The authors declare that they have no competing interests.

## Authors’ contributions

PG designed and programmed the machine learning method, participated in data analysis and participated in writing the manuscript. MK designed and supervised the study, participated in the method developments, data analysis and drafted the manuscript. Both authors read and approved of the final manuscript.

## Supplementary Material

Additional file 1 Full experimental dataset used in FISH Amyloid and classification results of 3 computational methods. Click here for file
